# Hydrophilic compounds in liquids of enzymatic hydrolyzed spruce and pine biomass

**DOI:** 10.1016/j.dib.2015.08.026

**Published:** 2015-09-03

**Authors:** Heli Sirén

**Affiliations:** Department of Chemistry, University of Helsinki, 00014 Helsinki, Finland

## Abstract

Organic acids are used for starting compounds in material sciences and in biorefinery, food, fuel, pharmaceutical, and medical industry. Here, we provide the data from a biochemical approach made to investigate production of organic acids and isolation of metals from wood, which is the most abundant biomass. Spruce and bark, phloem, and heartwood from pine were fermented with either microbes of oyster mushroom (*Pleurotus ostreatus)*, baker's yeast, or lactic acid bacteria to improve selective fermentation.

Using capillary electrophoresis and liquid chromatography techniques, we identified 14 different organic acids and phenolic acids with good yields. With inductively coupled plasma atomic emission spectroscopy 11 metals were quantified and further detailed analysis/results from these data are available in Sirén et al. (2015) [1].

**Specifications Table**

**Table:** 

Subject area	*Chemistry*
More specific subject area	*Biomaterials, biocompounds, forest chemistry*
Type of data	*Tables, text files, figures*
How data was acquired	*Two capillary electrophoresis instruments (P/ACE MDQ capillary electrophoresis, SCIEX, Beckman Coulter, Fullerton, CA, USA) both coupled with a photodiode array detector (PDA).*
*Two liquid chromatograph instruments (1260 Infinity Quaternary LC System and 1100 LC System, Agilent Technologies, Waldbronn, Germany) both on-line coupled with a diode array detector (DAD, HP 1100 LC).*
*The gas chromatograph – mass spectrometer instrument (GC–MS/MS instrument, Agilent Technologies 7890* *A GC System) coupled with an Agilent Technologies 7000 GC/MS Triple Quad (Agilent Technologies, Waldbronn, Germany). The Agilent Technologies 7693 Autosampler, the MassHunter Workstation Software, and the NIST Mass Spectral Search Program 2.0.*
*An ICP-AES instrument (IRIS Interpid II XDL (Thermo Fisher Scientific, Vantaa, Finland).*
*Microwave-assisted extraction (MAE) instrument (Anton PAAR, USA).*
*Three different bioreactors (vol. 0.5* *L, 2* *L, and 3.5* *L (laboratory-made instruments).*
Data format	*Raw data*
Experimental factors	*Organic compounds were produced from wood in the bioreactors with either microbes of oyster mushroom (P. ostreatus), baker’s yeast, or lactic acid bacteria. The compounds were analyzed straight from the fluids. Metals were isolated with MAE.*
Experimental features	*The samples are pieces from logs of living trees. They were pretreated with a gentle fermentation.*
Data source location	*Helsinki, Finland and Lappeenranta, Finland*
Data accessibility	*The data are with this article.*

**Value of the data**

• New route for production of chemicals from softwood can be used for polymers and biomaterials
• Green chemistry production for materials with microbes can be applied for manufacturing biodegradable products and cleantech materials
• Processes are more widely applicable for integration to biorefinery
• The diverse separation technology for water-soluble bioproducts will be useful for other researchers who are developing food preservatives, hygienic products, antibacterial products and medicines for self-care drugs.

## 1 Data

The data shown is schemes and a photograph explaining details of pretreatment and microreactor devices used to process the pine materials. The chromatograms, electropherograms, histograms, and the table show the profiling of the biofluids, the efficiency of the Oyster mushroom against some other microbes, and the quantities of the most important acids in hydrolyzed heartwood, bark, and phloem of pine.

## 2 Experimental design and materials

**Fig.1:**
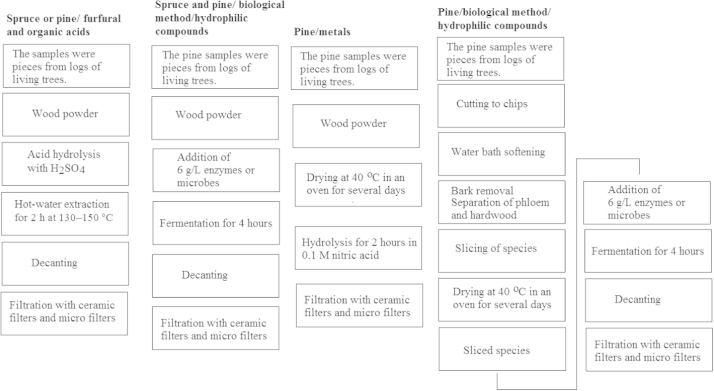
Overall workflow of the samples.

Hydrophilic organic acids in fresh spruce and pine wood extracts were studied after processing them in bioreactors in the presence of microbes from oyster mushroom (*Pleurotus ostreatus*) or enzymes from dried yeast (*Saccharomyces cerevisiae*). The organic acids were extracted from sorted-out wood material. Metals were also studied, but only after the wood was oven-dried. In the case of no dried samples, the purpose was to use the natural microbes of the fresh trees (Fig. 1).

## 3 Methods

**Fig. 2:**
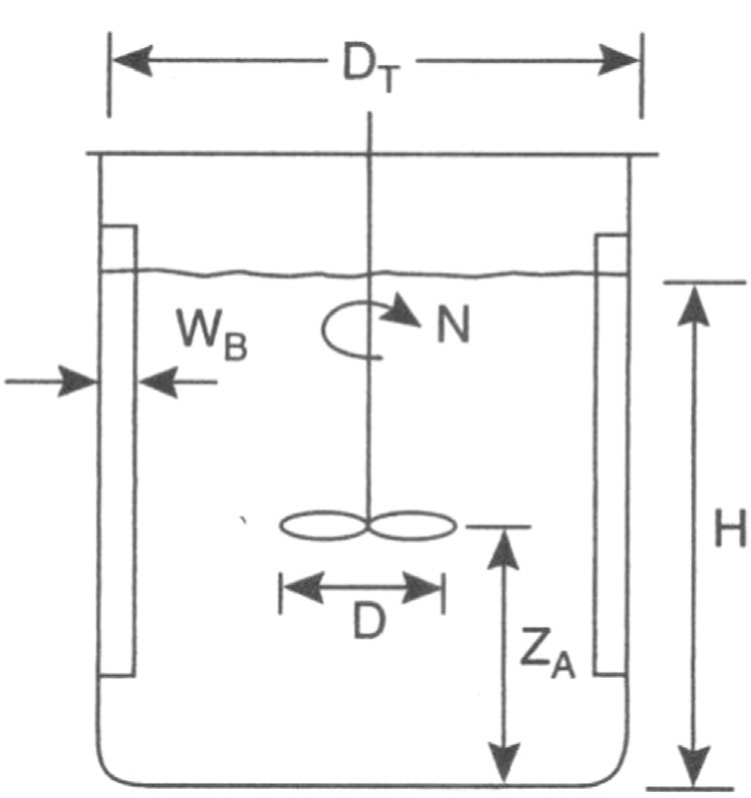
The dimensions of the bioreactor in the final results. The height of the reactor was 24.9 cm. Diameter of the reactor (*D*_T_) was 16.4 cm and the diameter of the mixer (*D*) was 10.2 cm. In bark extraction distance of the mixer from the bottom (*Z*_A_) was 5.4 cm. In the other extraction *Z*_A_ was 4.3 cm, except in the first extractions, in which the distance of the mixer from the bottom was 4 cm. *N* is the rotor. *H* is the distance between the liquid level and the bottom of the mixer. *W*_B_ is the thickness of the polytetrafluoroethylene polymer (PTFE) coating surface in the reactor.

**Fig. 3:**
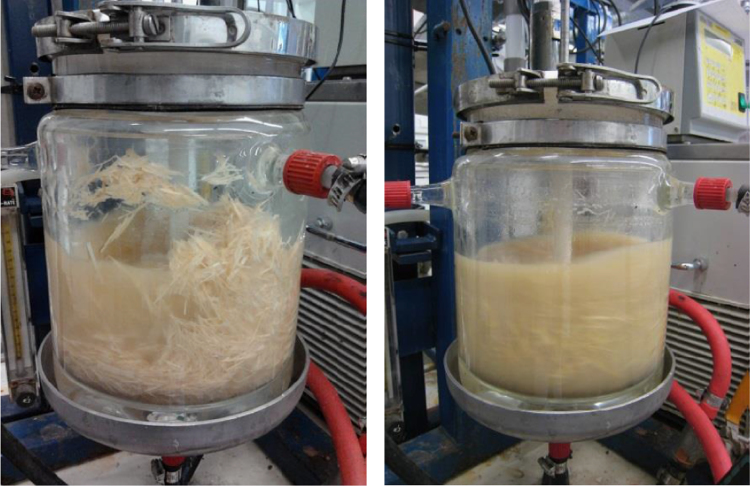
Enzyme hydrolysis start (above) and stop (below). Sample: chipped softwood of pine. Volume of the bioreactor: 3.5 L; Solution: 2 L water; Solid/liquid ratio 40; Mixing rate 250 rpm; Temperature 30 °C; Pressure 2 bar. (*Photographs by Päivi Riikonen*).

The methods used were ICP-AES, inductively coupled plasma atomic emission spectroscopy; CE, capillary electrophoresis; UV, ultraviolet spectrophotometry; HPLC, high-performance liquid chromatography; DAD, diode array detection; MAE, microwave-assisted extraction; bioreactor fermentation (Figs. 2 and 3); acid hydrolysis.

**Fig. 4:**
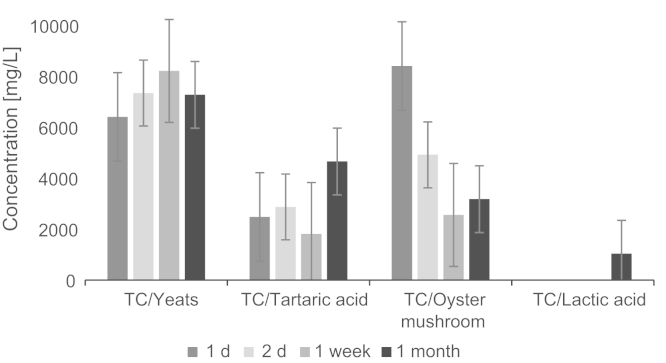
Comparison of fermentation microbes based on extraction time using total carbon concentration (TC). Fermentation with yeast and tartaric acid were used to compare their ability to that of oyster mushroom. The two first are used in well-known wine fermentation and ethanol production. Oyster mushroom worked efficiently only in the first day for acid formation [1].

**Fig. 5:**
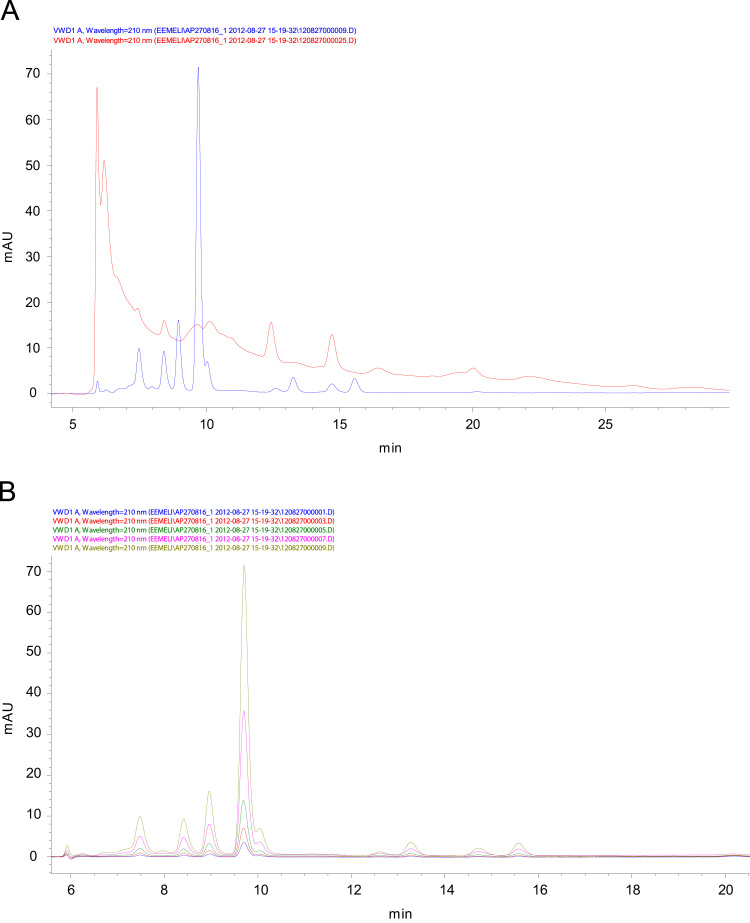
A. Chromatogram of 100 mg/L standard mixture (blue). Spruce–water–yeast extract (red) made at 55 °C, 1 bar for 2 h. The extract is taken from the bioreactor without dilution or filtration. A Varian MetaCarb 87 H column (Agilent Technologies Finland Oy, Espoo, Finland) with polystyrene sulfonate resin (8–10 µm, at 8% of graft) was used. Its dimensions (length x i.d.) were 300×7.8 mm^2^. The mobile phase was 0.005 M H_2_SO_4_ made in ultra-pure water. Its flow rate was 1.0 mL/min. The compounds were detected with UV at 210 nm. B. Chromatograms of 50, 25, 15, 7, 4, and 2 mg/L standards mixtures. Peaks in the elution order: 1. Ascorbic acid, 2. citric acid, 3. tartaric acid, 4. pyruvic acid, 5. D-malic acid, 6. L-lactic acid, 7. D-lactic acid, 8. L-malic acid, and 9. acetic acid. The column was a Luna 100×3.00 mm (C18, 5 µm) column from Phenomenex. The mobile phase was made of 0.1% formic acid in water and methanol. Gradient elution was made from 7% methanol to 9% in 15 min and to 60% in the next 35 min. The flow rate of the mobile phase was 0.5 mL/min. The UV detection was at 210 nm. Separation temperature was 30 °C. Sample amount was 10 μL. (For interpretation of the references to color in this figure caption, the reader is referred to the webversion of this paper.)

**Fig. 6:**
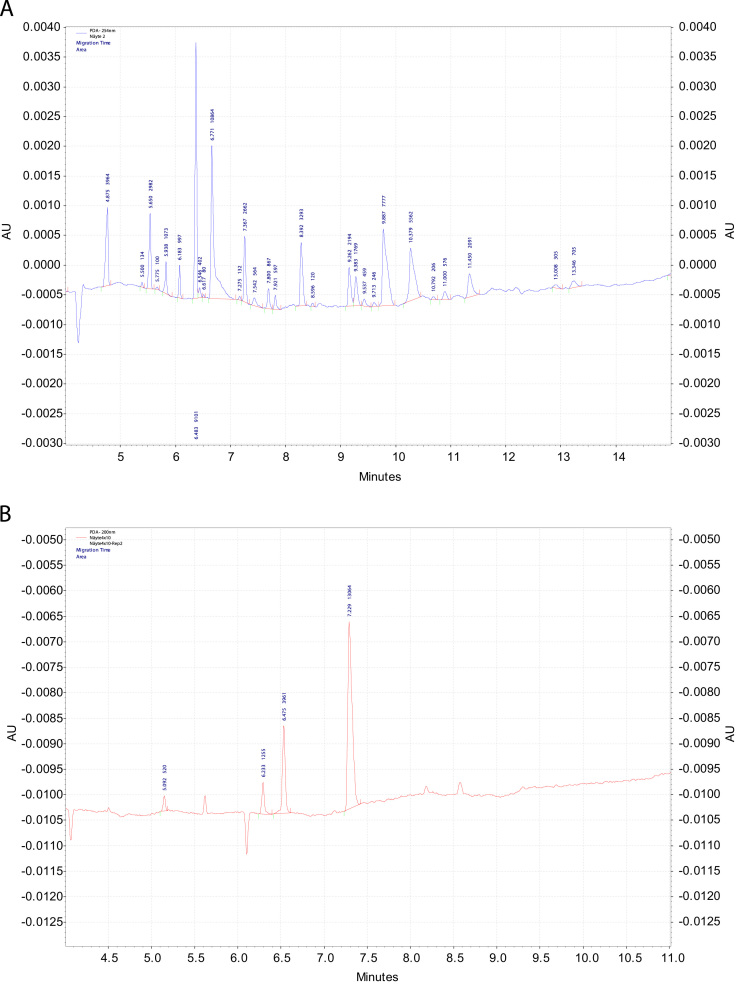
A. Electropherogram of a hydrolysate of spruce sample. The electrolyte solution in separation of phenolic acids was prepared from 0.2 M N-cyclohexyl-3-aminopropanesulfonic acid (CAPS) to get a solution composition of 60 mM CAPS and 40 mM NaOH at pH 10.7. The phenolic acids were detected directly at 254 nm wavelength.. In that case the separation voltage was 20 kV (normal polarity). Samples were injected at 1 p.s.i. pressure for 8 seconds. B. Electropherogram of a processed sample. Identified peaks from the left: formic acid, adipinic acid, acetic acid, and propionic acid. The organic acids without aromatic rings were separated in an electrolyte solution, which contained 20 mM 2,3-pyrazinedicarboxylic acid (2,3 PDC), 0.3 mM MTAH, and 30 mg/L of both Ca^2+^ and Mg^2+^ in MeOH–water solution (10:90, v/v) at pH 9.0.

The bioreactor process was optimized for extraction of hydrophilic organic acids in fresh spruce and pine wood. The products were processed at the presence of microbes from oyster mushroom (*P. ostreatus*) or alternatively with enzymes from dried yeast (*S. cerevisiae*) (Fig. 4). The wood materials were sorted-out from softwood. The fluids contain innumerable amounts of various kinds of organic compounds. Because of the similarity of their structures, the top value-added organic acids and chemicals need to be separated (Figs. 5 and 6) for identification and quantification.

Metals were also studied from oven-dried woods. In the case of non-dried samples the purpose was to use the natural microbes of the fresh trees and to minimize the material costs. First of all, the added microbes needed to work in fast degradation of lignin, hemicelluloses, cellulose, and polysaccharides.

**Fig. 7:**
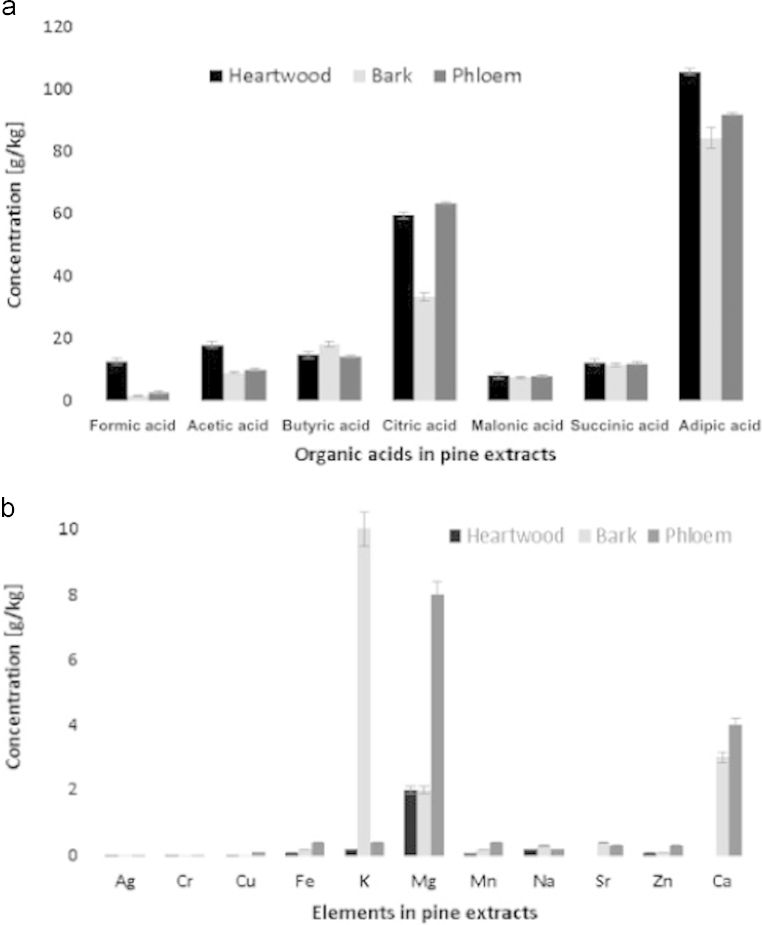
Main organic acids and metals in the heartwood, bark, and phloem of pine: (A) organic acids with CE. The organic acids without aromatic rings were separated in an electrolyte solution, which contained 20 mM 2,3-pyrazinedicarboxylic acid (2,3 PDC), 0.3 mM MTAH, and 30 mg/L of both Ca^2+^ and Mg^2+^ in MeOH–water solution (10:90, v/v) at pH 9.0; (B) metals with ICP-EAS. [1].

The acids were mainly localized in both skin and pulp of the wood (Fig. 7). Table 1 also provides an overview of the main organic acids in the fermentation processes of spruce and pine by CE and HPLC.

## Figures and Tables

**Table 1:** Production of the main organic acids in fermentation processes of spruce and pine analyzed with CE and HPLC. [1].

**Organic acid**	**Processed (g/kg)**	**Wood type**
L-Lactic acid (*S. cerevisiae*)	0.36	Mix, spruce
L-Malic acid (*S. cerevisiae*)	0.45	Mix, spruce
Succinic acid (*P. ostreatus*)	0.89	Bark+phloem+heartwood, pine
Adipic acid (*P. ostreatus*)	7.05	Bark+phloem+heartwood, pine
Citric acid (*P. ostreatus*)	3.61	Bark+phloem+heartwood, pine
